# Trends in Rhinoplasty Research in Saudi Arabia: A 30-Year Bibliometric Analysis

**DOI:** 10.7759/cureus.67619

**Published:** 2024-08-23

**Authors:** Mohammed Khan, Abdularhman Alnefaie, Wedyan H Almosa, Walaa Sherhri, Mohammed Alhejaily, Ahmed Alarfaj

**Affiliations:** 1 Department of Otolaryngology - Head and Neck Surgery, King Abdullah Medical City, Makkah, SAU; 2 Department of Otolaryngology - Head and Neck Surgery, Prince Mansour Military Hospital, Jeddah, SAU; 3 Department of Otolaryngology - Head and Neck Surgery, Alnoor Specialist Hospital, Makkah, SAU; 4 Department of Otolaryngology - Head and Neck Surgery, King Abdulaziz University, Riyadh, SAU; 5 Department of Otolaryngology - Head and Neck Surgery, Ohud Hospital, Medinah, SAU; 6 Department of Otolaryngology, King Abdulaziz University Hospital, Riyadh, SAU

**Keywords:** publications, trends, bibliometric analysis, saudi arabia, rhinoplasty

## Abstract

Rhinoplasty is one of the most commonly performed aesthetic surgeries. Saudi Arabia has a large number of rhinoplasty publications. Here, we aimed to assess the past, present, and future research output related to rhinoplasty in Saudi Arabia and compare it with global output. We performed a bibliometric analysis to evaluate rhinoplasty research productivity trends in Saudi Arabia from 1995 to 2021 in both cosmetic and reconstructive rhinoplasties. We considered all publications whose first authors were from Saudi Arabia or whose authors contributed significantly to a paper from Saudi Arabia, even though the first author was not from the Kingdom of Saudi Arabia. We evaluated the research quality of the articles using the impact factor of the journal in which the article was published. For each article, the number of authors, number of citations received, study design, region of the first author, and the level of evidence were collected. We gained several insights into the global trends of rhinoplasty in research and its position. We observed a significant increase in the number of publications on rhinoplasty in Saudi Arabia. Although there was no significant increase in the impact factor, most publications had a level of evidence of I or II.

## Introduction and background

Rhinoplasty is one of the most commonly performed aesthetic surgeries worldwide. It was the most-performed cosmetic procedure in 2020 in the United States according to the American Society of Plastic Surgeons National Clearinghouse of Plastic Surgery Procedural Statistics [1]. Rhinoplasty was the second most commonly published topic in a study by Chang et al., 2017, that evaluated the 50 most cited articles on facial plastic and reconstructive surgery [2]. In a recent study by Lalezari et al., 2018, that performed a bibliometric analysis and evaluated the research productivity trends in rhinoplasty, the United States was the leading country in research publication volume in rhinoplasty, followed by Asian countries, and Eastern European countries were the least contributing region [3]. A bibliometric analysis study by Sinha et al., 2016, evaluated the 100 most cited articles on rhinoplasty, and the results showed that plastic surgery journals were the highest contributors to the top 100 articles, followed by otolaryngology/ear, nose, and throat [4]. Most of the 100 most cited articles on rhinoplasty were published in Plastic and Reconstructive Surgery followed by Archives of Otolaryngology-Head and Neck Surgery [4].

Saudi Arabia has a large number of rhinoplasty publications. Alharethy et al. conducted a cross-sectional study to investigate the demographic characteristics of Saudi patients seeking cosmetic procedures at three different private hospitals in two different regions in Saudi Arabia in 2017. The author found that a typical Saudi cosmetic surgery patient is a university graduate, married, employed, and middle-aged (20-40 years of age), with a fairly high typical monthly income [5].

In the present study, we aimed to assess the past, present, and future of research output related to rhinoplasty in Saudi Arabia and compare it with the global output.

## Review

Materials and methods

Study Design and Population

We performed a bibliometric analysis to evaluate rhinoplasty research productivity trends in Saudi Arabia from 1995 to 2021 in both cosmetic and reconstructive rhinoplasties. Ethical approval from the review board was obtained before starting the study. We considered all publications during this period whose first authors were from Saudi Arabia or whose authors contributed significantly to a paper from Saudi Arabia, even though the first author was not from the Kingdom of Saudi Arabia. We evaluated the research quality of the articles using the impact factor (IF) of the journal in which the article was published [6]. For each article, the number of authors, number of citations received, study design, region of origin of the first author, and the level of evidence (LOE) were collected. We distinguished the research articles based on whether the research was primarily aesthetic or reconstructive in nature. We also considered the subspecialties of the studies.

Literature Search

In May 2021, the following search engines were used to retrieve the articles: Web of Science, Google Scholar, Cochrane, and Proquest. All Saudi-affiliated articles were included in this study. Articles published until 2021 were selected from English-language journals. All selected articles were either peer-reviewed original articles or case reports. Articles contributing to the field of rhinoplasty, such as non-surgical rhinoplasty and anesthesia, were also included. Editorial letters, conference abstracts, and book reviews; duplicate entries after careful inspection of the list; and articles related to only septoplasty were excluded.

IFs for journals from 1995 to 2021 were compiled from the Web of Science Journal Citation Reports [7]. The IF was calculated by dividing the total number of citations a journal receives in a particular year by the number of articles published by the journal in the previous two years. A few of the journals were not assigned an IF for a particular year during the study period; therefore, articles published in a journal lacking an IF for the corresponding year were excluded from the analysis involving IF or productivity index.

Classification of Articles

All articles were classified as either clinical or basic. Clinical research articles were further classified as prognostic or therapeutic in line with previously published guidelines [8]. The self-reported LOE was used when available. For clinical studies without a self-reported LOE, a rating was assigned according to the published American Society of Plastic Surgeons (ASPS) Evidence Rating Scale [9]. The productivity index was calculated for each year by multiplying the total number of articles published in that year with the IF of the journal containing each article for the given year [10].

The type of study within rhinoplasty literature was further distinguished as a clinical or basic science research article. Each clinical research article was assigned a type of investigation as either aesthetic rhinoplasty or reconstructive rhinoplasty. The criteria for reconstructive rhinoplasty included nasal reconstruction associated with cleft deformity or other congenital defects and rhinoplasty to repair a defect caused by trauma or removal of a tumor or other growth. All other clinical articles were considered aesthetic studies.

Statistical Analysis

Data are presented as mean ± standard deviation. Trends were analyzed using linear regression analysis, and p-values were reported based on associated t-tests for the regression coefficients. Statistical analyses were performed using IBM SPSS Statistics for Windows, Version 28, (Released 2021; IBM Corp., Armonk, New York, United States). The significance level was set at α=0.05.

Results

Literature Search Results

Eighty research papers were published from 1995 to 2021 (Appendix 1). In 2015, there was an increase in rhinoplasty research, with a maximum of 21 studies published in 2020.

Publication Volume and Productivity Index

**Figure 1:**
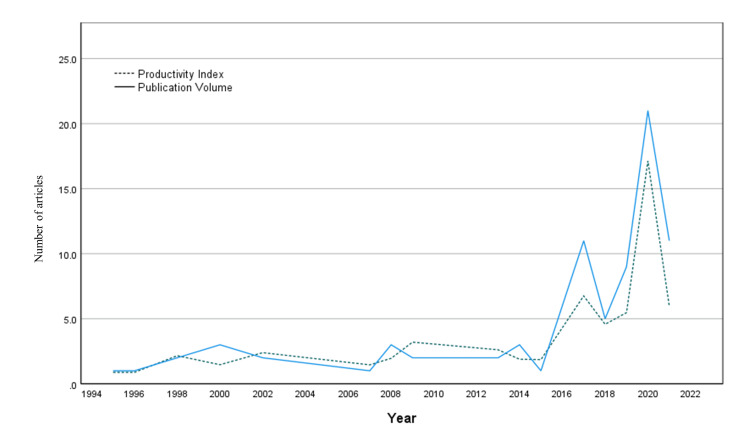
Volume and productivity of rhinoplasty research in Saudi Arabia from 1995 to 2021.

The overall average number of publications per year was 4.71 ± 5.38. There was a significant increase in publication volume of 0.39 ± 0.12 articles per year (p=0.006, R2=0.40). The total productivity index of rhinoplasty articles from Saudi Arabia over the study period was 60.68, with a mean productivity index of 3.79 ± 1.00, which increased significantly by 0.27±0.094 per year (p=0.012, R2=0.373). Figure 1 shows the publication volume per year and the productivity index over time.

Journals

The top three journals in terms of rhinoplasty publishing volume were Saudi Medical Journal (eight articles, 10.0%), Cureus (five articles, 6.3%), and Plastic and Reconstructive Surgery Global Open (four articles, 5.0%). All journals that published one or two articles during this period were grouped in the other category, and there were 40 such journals (66.25% of the articles). Table 1 shows the distribution of articles published in different journals.

**Table 1:** Characteristics of included studies

Item	N	%
Journal		
Eur Arch Otorhinolaryngology	3	3.75 %
Ear Nose Thorat J	3	3.75 %
Ann Saudi Med	4	5%
Plast Reconstr Surg Glob Open	4	5%
Cureus	5	6.25%
Saudi Med J	8	10%
Others	53	66.25%
Study design		
Prospective cohort	24	30%
Retrospective cohort	22	28.75%
Case series	14	17.5%
Cross-sectional	8	10%
Clinical trials	3	3.7%
Review	3	3.7%
Meta-analysis	3	3.7%
Systematic review	3	3.7%
Study subtype		
Therapeutic clinical study	39	48.70%
Prognostic clinical study	31	38.70%
Basic science study	10	12.50%
Subspecialty		
Otolaryngology	60	75%
Plastic surgery	10	12.5%
Maxillofacial	7	8.75%
Anesthesia	2	2.5%
Dermatology	1	1.25%
Region of the first author		
Riyadh	50	62.5%
Jeddah	14	17.5%
Makkah	5	6.25%
Najran	2	2.5%
Qassim	2	2.5%
Others	7	8.75%

Study designs, subtypes, and subspecialties

More than half of the studies were either prospective (30%) or retrospective studies (28.75%); case series and cross-sectional studies constituted 17.5% and 10%, respectively. Meanwhile, the percentage for clinical trials, reviews, meta-analyses, and systematic reviews was 3.7% each. Table 1 shows the frequencies of different study designs.

The majority of articles over the study period were classified as clinical (70 articles, 87.5%), and basic science articles were a minority (10 articles, 12.5%). In the clinical research rhinoplasty literature, 39 articles were classified as therapeutic and 31 articles as prognostic (Table 1).

Regarding the subspecialty of studies, the majority of the publications were in the subspecialty of otolaryngology with 60 articles (75.0%), followed by 10 articles in plastic surgery with 12.5% of the total publications. Only one publication was published in dermatology. Among the remaining publications, seven were published in the maxillofacial field and two were in the field of anesthesia (Table 1).

Region of the First Author

Most publications on rhinoplasty (N=50, 62.5%) had a first author from Riyadh, followed by 14 (17.5%) publications with a first author from Jeddah. There were five articles whose first author was from Makkah. There were two articles each from Najran and Qassim, respectively. There were seven other cities with one publication from each city (Table 1).

IF

**Figure 2:**
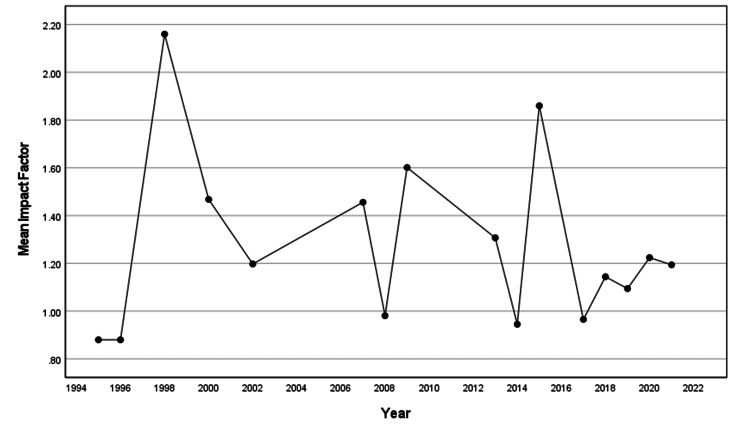
Mean impact factor for the publications on rhinoplasty from Saudi Arabia for each year of publication

Over the study period, the mean IF for the articles published did not increase significantly (β=-0.004, standard error (SE)=0.011, p=0.687, R2=0.012). Due to the lack of available IFs for a given year, 29 publications were excluded from the analysis. The overall mean IF of the journals where these 51 articles were published was 1.19 ± 0.51. (Figure 2).

Aesthetic Rhinoplasty Versus Reconstructive Rhinoplasty

Most rhinoplasty publications were classified as aesthetic (58 articles, 72.5%). Reconstructive articles comprised 16.25% (13 articles) of the Saudi Arabian rhinoplasty literature, and there were seven articles (8.75%) in the basic sciences.

Over the study period, the annual volume of publications on aesthetic rhinoplasty significantly increased by 0.221 ± 0.072 articles per year (p=0.008, R2=0.384). The annual number of publications on reconstructive rhinoplasty did not increase significantly over the years (β=0.119, SE=0.065, p=0.088, R2=0.181).

In addition, when evaluating the proportion of articles published in a given year as being aesthetic, reconstructive, or basic science in nature, there were no significant changes in basic (p=0.13, R2=0.149), aesthetic (β=-0.015, SE=0.004; p=0.004; R2=0.435), or reconstructive rhinoplasty (β=0.008, SE=0.003; p=0.022; R2=0.303).

Number of Authors

**Figure 3:**
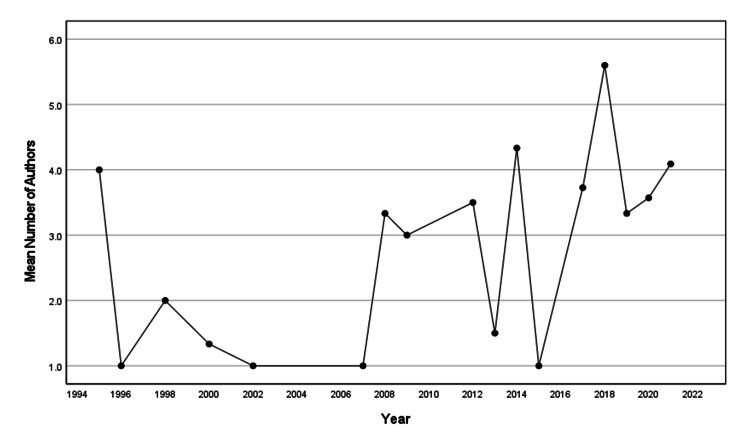
Mean number of authors by year.

The mean number of authors for an article was 3.44 ± 2.33 over the whole study period. Figure 3 shows a plot of the mean number of authors over time. The mean number of authors increased significantly over time (β=0.083, SE=0.037, p=0.041, R2=0.249).

Number of Citations

**Figure 4:**
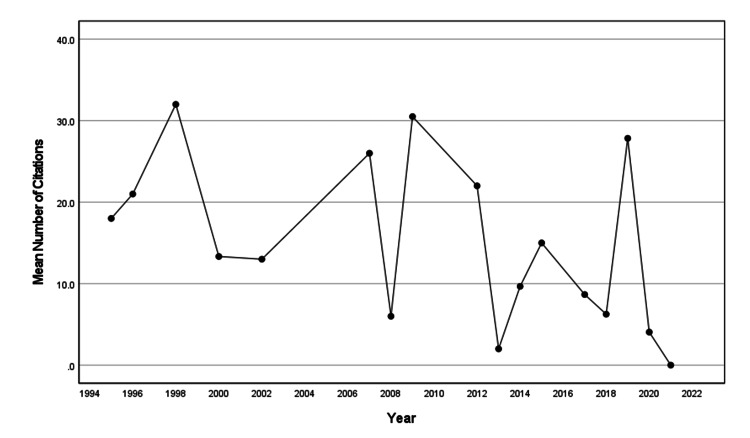
Mean number of citations for a publication by year.

The total number of citations of the 62 articles in this study was 679. The average number of citations for an article was 10.95±15.77 with one article having 97 citations. Figure 4 shows the mean number of citations per article over the years, which did not change significantly over time (β=-0.538, SE=0.268, p=0.064, R2=0.211).

LOE

There were 21 studies with level I (26.3%), 23 studies with level II (28.7%), 13 studies with level III (16.3%), three studies with level IV (3.8%), and 12 studies with level V (15.0%) articles. There were eight articles for which we did not have any LOE.

During the study period, there was a significant increase in level II (β=0.14, SE=0.05, p=0.009, R2=0.379) and level V studies (β=0.083, SE=0.029, p=0.012, R2=0.353) each year. There were slight increases in level I (β=0.039, p=0.247), level III (β=0.048, p=0.088), and level IV studies (β=0.23, p=0.131) each year, but these increases were not statistically significant.

As we have observed before, most of the research in rhinoplasty was from the region of Riyadh; most of the level I (81%), level II (60.9%), level III (53.8%), and level V (50.0%) studies were from Riyadh. However, Jeddah had a high volume of level III (38.5%) and level IV studies (66.7%).

Discussion

Rhinoplasty is one of the most popular plastic surgery procedures worldwide [3]. Here, we presented the results of a bibliometric analysis of all rhinoplasty articles published between 1995 and 2021, where researchers from Saudi Arabia made a significant contribution. According to the article by Lalezari et al., 2018 [3], we found a significant increase in the volume of publications on rhinoplasty worldwide, and the IF of journals where these articles were published also increased globally. Similarly, we observed a significant increase in the volume of publications on rhinoplasty in Saudi Arabia. While the global rate of increase reported in Lalezari et al.’s 2018 study was 3.5 articles per year, the rate of increase of articles per year from Saudi Arabia was 0.39, which is quite commendable compared with other developing nations [3]. Given the recent bibliometric research in Ri et al.’s 2022 study, we observed that the total number of publications on rhinoplasty during 2020-2021 is comparable to the number of publications in Japan and France; however, Ri et al.’s study did not give the actual numbers for comparison [11].

It is also worth noting that the global productivity index for all the regions considered in Lalezari et al.’s study was 85.5± 54.3 for the period 1993-2013, but the productivity index for Saudi Arabia alone was 60.68 during 1995-2021 [3]. The regions considered in Lalezari et al.’s study are much larger than those in Saudi Arabia. Although the numbers are not strictly comparable due to differences in the regions and the time periods considered, it shows that rhinoplasty research is not far behind globally in terms of the volume of publications and quality of publications as measured by Lalezari et al., 2018 [3].

Our bibliometric analysis showed that 61.1% of the total publications had an LOE of I or II, while only approximately 21% of the articles published had an LOE of IV or V. This is in contrast with that reported in the global rhinoplasty literature, where about 76% of all articles in rhinoplasty have LOEs of IV or V. However, there has been a significant increase in the number of publications with LOEs of III and V over the years. Publications with LOEs I, II, and IV also showed an upward trend.

Although there has been a global increase in the IF of journals for rhinoplasty publications, we did not observe any significant increase in the IF or productivity index for publications from Saudi Arabia. Although most publications were clinical in nature, they were either therapeutic or prognostic. No publications were diagnostic in nature. Additionally, most of the articles were of an aesthetic nature rather than a reconstructive nature, and only a few were in basic sciences. The number of articles on aesthetic and reconstructive rhinoplasties has increased significantly over the years.

The average number of authors for an article increased significantly over time, indicating that more people are collaborating on research projects, which is a positive indicator of the research culture in the country. The number of citations is also an indicator of the quality of an article and its impact on further research. We did not observe any significant change in the number of citations. In addition, we would like to note that recent articles were more likely to have a smaller number of citations than older articles. Although we believe that the quality and quantity of research have started increasing recently, this is not reflected in the number of citations, as not enough years have passed by to have a large number of citations.

For these regions, we observed that most of the research originated in Riyadh, followed by Jeddah. This finding shows the dominance of a single research group based in Riyadh for rhinoplasty research in Saudi Arabia. However, it is good to know that other research groups are also slowly emerging in this area.

Otolaryngology, with 60 articles (75.0%) had the majority of publications, followed by plastic surgery with 10 articles (12.5%). There was limited contribution from the dermatology, maxillofacial, and anesthesia fields.

According to a recent comprehensive summary of the publications on rhinoplasty from 2012 to 2021, the United States was the largest contributor to rhinoplasty research, followed by Turkey. Other major contributors were Korea, China, and Iran. However, most aesthetic research focused on augmentation rhinoplasty, which is highly performed by Asians. Also, most of the studies were dependent on self-reported outcome evaluation scales [12].

In this bibliometric analysis of rhinoplasty publications in Saudi Arabia, we gained several insights into the global trends of rhinoplasty in research and its position. However, we must acknowledge several limitations. First, we considered publications only in English, which may have underestimated total research productivity. Second, although IF is widely accepted and used in the scientific community as a measure of quality, it has flaws and is susceptible to manipulation [13]. Lastly, we excluded 29 articles because of a lack of information on IFs, which may have affected the accuracy of the analysis of the mean IF and productivity index over the years.

## Conclusions

In conclusion, we observed a significant increase in the number of publications on rhinoplasty in Saudi Arabia. Although there was no significant increase in the IF, most publications had an LOE of I or II. We also gained insight into the productivity index, the volume of publications, most contributing regions, most published authors, number of authors, number of citations received, study design, IF, most published specialty, and LOE of publications. The results of this analysis could act as a benchmark for future studies, help resource allocation within the Kingdom of Saudi Arabia, and promote further advancement in rhinoplasty research.

## Appendices

**Table 2:** List of included studies

Number	Citation
1	Gelidan AG. Technique to Fix the Stubborn Deviated Caudal Septum with an Internal Bone Graft Splint. Plast Reconstr Surg Glob Open. 2021 Dec 20;9(12):e3921. doi: 10.1097/GOX.0000000000003921. PMID: 34938641; PMCID: PMC8687722.
2	Abdulrauf BMI. Cleft Rhinoplasty: A Tug of War. Plast Reconstr Surg Glob Open. 2021 Oct 26;9(10):e3839. doi: 10.1097/GOX.0000000000003839. PMID: 34712542; PMCID: PMC8547928.
3	Mulafikh DS, Alharethy SE, Alqabbani AA, Mesallam TA. Validation and clinical application of the Arabic rhinoplasty outcomes evaluation questionnaire. Saudi Med J. 2021 Jun;42(6):655-659. doi: 10.15537/smj.2021.42.6.20210038. PMID: 34078728.
4	Shah Mardan QNM, Mrad MA, Khojah AA, Hashem FK. Rhinoplasty for Hypertrophied Transverse Nasalis Muscle: A Case Report. Plast Reconstr Surg Glob Open. 2021 May 24;9(5):e3593. doi: 10.1097/GOX.0000000000003593. PMID: 34046293; PMCID: PMC8143773.
5	Abdulrauf BMI. The nasal cantilever technique in children undergoing primary cleft lip surgery: novel concepts and review. Innov Surg Sci. 2020 Sep 7;5(1-2):53-62. doi: 10.1515/iss-2020-0016. PMID: 33506094; PMCID: PMC7798302.
6	Abdulrauf BMI. The Nasal Lift Technique in Primary Cleft Lip Surgery. Cleft Palate Craniofac J. 2021 Jul;58(7):919-924. doi: 10.1177/1055665620964708. Epub 2020 Oct 16. PMID: 33063522.
7	Zeid NG, Mohamed AS, ElSayed ElFouly M, Azooz KO, Aleryan MM, Abd Elmottaleb Sabaa M. Objective Comparison Between Spreader Grafts and Flaps for Mid-Nasal Vault Reconstruction: A Randomized Controlled Trial. Plast Surg (Oakv). 2020 Aug;28(3):137-141. doi: 10.1177/2292550319880919. Epub 2019 Oct 30. PMID: 32879868; PMCID: PMC7436848.
8	Alghonaim Y, Arafat AS, Alobaid F. Postoperative malodorous smell in open septorhinoplasty: the effect of intradomal suturing with mucosal release. Eur Arch Otorhinolaryngol. 2021 Mar;278(3):703-709. doi: 10.1007/s00405-020-06307-x. Epub 2020 Aug 27. PMID: 32856122.
9	Hudise JY, Aldhabaan SA, Nassar RS, Alarfaj AM. Evaluation of Scar Outcome After Alar Base Reduction Using Different Surgical Approaches. J Oral Maxillofac Surg. 2020 Dec;78(12):2299.e1-2299.e8. doi: 10.1016/j.joms.2020.06.021. Epub 2020 Jun 20. PMID: 32668196.
10	Alqabbani AA, Alateeq MF, Alshalaan Z. Isolated Congenital Supernumerary Nostril in an Adult Patient. Ear Nose Throat J. 2021 Dec;100(10_suppl):1038S-1040S. doi: 10.1177/0145561320933390. Epub 2020 Jun 18. PMID: 32551959.
11	Hudise JY, Aldhabaan SA, Alwadani MM, Alqabbani AA, Bafaqeeh SA. Sail excision technique for overhanging thick ala in Saudi Arabia. Saudi Med J. 2020 Jun;41(6):635-639. doi: 10.15537/smj.2020.6.25117. PMID: 32518931; PMCID: PMC7502936.
12	Ali YH, Alandejani T. Rhinoplasty Assisted by Endoscopic Septoplasty: Precise Job and an Educational Tool. J Craniofac Surg. 2020 May/Jun;31(3):847-850. doi: 10.1097/SCS.0000000000006117. PMID: 32168123.
13	Alkarzae M, Bafaqeeh SA. Turn-in Flap: 10 Years' Experience of a Single Institution in Saudi Arabia. Cureus. 2020 Jan 7;12(1):e6593. doi: 10.7759/cureus.6593. PMID: 32051804; PMCID: PMC7001133.
14	Jomah J, Elsafi RA, Ali KSAE, Abdullah R, Gelidan AG. Nasal Skin Thickness Measurements Using Computed Tomography in an Adult Saudi Population. Plast Reconstr Surg Glob Open. 2019 Sep 30;7(9):e2450. doi: 10.1097/GOX.0000000000002450. PMID: 31942407; PMCID: PMC6908396.
15	Mirza AA, Alandejani TA, Al-Sayed AA. Piezosurgery versus conventional osteotomy in rhinoplasty: A systematic review and meta-analysis. Laryngoscope. 2020 May;130(5):1158-1165. doi: 10.1002/lary.28408. Epub 2019 Nov 22. PMID: 31758577.
16	Aldosari B. Is Nasal Skin Thickness a Prognostic Indicator to Postoperative Edema and Ecchymosis? Ear Nose Throat J. 2021 May;100(4):NP206-NP209. doi: 10.1177/0145561319868452. Epub 2019 Sep 29. PMID: 31566001.
17	Aldihan K, Alnasyan A, Albassam A, Alghonaim Y, Aldekhayel S. Comparing the Health Burden of Living With Nasal Deformity in Actual Patients and Healthy Individuals: A Utility Outcomes Score Assessment. Ann Plast Surg. 2019 Oct;83(4):381-383. doi: 10.1097/SAP.0000000000002065. PMID: 31524727.
18	Mrad MA, Almarghoub MA. Skin Necrosis following Rhinoplasty. Plast Reconstr Surg Glob Open. 2019 Feb 8;7(2):e2077. doi: 10.1097/GOX.0000000000002077. PMID: 30881829; PMCID: PMC6416127.
19	Aljabaa AH. Lateral cephalometric analysis of the nasal morphology among Saudi adults. Clin Cosmet Investig Dent. 2019 Jan 15;11:9-17. doi: 10.2147/CCIDE.S190230. PMID: 30679927; PMCID: PMC6338237.
20	Alharethy S, Mousa A, Alharbi A, Aldrees T, AlQaryan S, Ju Jang Y. Does skin thickness affect satisfaction post rhinoplasty? Middle Eastern population as an example. Saudi Med J. 2018 Dec;39(12):1238-1241. doi: 10.15537/smj.2018.12.23269. PMID: 30520507; PMCID: PMC6344664.
21	Jang YJ, Kim SA, Alharethy S. Failure of Synthetic Implants: Strategies and Management. Facial Plast Surg. 2018 Jun;34(3):245-254. doi: 10.1055/s-0038-1654676. Epub 2018 Jun 1. Erratum in: Facial Plast Surg. 2018 Aug;34(4):429-430. PMID: 29857334.
22	Alharethy S, Alohali S, Alquniabut I, Jang YJ. Bone and Soft Tissue Nasal Angles Discrepancies and Overlying Skin Thickness: A Computed Tomography Study. Aesthetic Plast Surg. 2018 Aug;42(4):1085-1089. doi: 10.1007/s00266-018-1127-9. Epub 2018 Apr 11. PMID: 29644414.
23	Alharethy SE. Trends and demographic characteristics of Saudi cosmetic surgery patients. Saudi Med J. 2017 Jul;38(7):738-741. doi: 10.15537/smj.2017.7.18528. PMID: 28674720; PMCID: PMC5556282.
24	AlHarethy S, Al-Angari SS, Syouri F, Islam T, Jang YJ. Assessment of satisfaction based on age and gender in functional and aesthetic rhinoplasty. Eur Arch Otorhinolaryngol. 2017 Jul;274(7):2809-2812. doi: 10.1007/s00405-017-4566-z. Epub 2017 Apr 17. PMID: 28417237.
25	Alharethy S. Preferred nasolabial angle in Middle Eastern population. Eur Arch Otorhinolaryngol. 2017 May;274(5):2339-2341. doi: 10.1007/s00405-017-4507-x. Epub 2017 Feb 27. PMID: 28243784.
26	Mirza AA, Marglani OA, Farooq MU, Al-Khatib TA, Jameel WS, Sultan NA, Aly MS. Comparison of two approaches to lateral nasal osteotomy in Saudi patients. Ann Saudi Med. 2017 Jan-Feb;37(1):42-48. doi: 10.5144/0256-4947.2017.42. PMID: 28151456; PMCID: PMC6148979.
27	Al Arfaj AM. The use of nasal packing post rhinoplasty: does it increase periorbital ecchymosis? A prospective study. J Otolaryngol Head Neck Surg. 2015 Jun 16;44(1):22. doi: 10.1186/s40463-015-0075-5. PMID: 26077040; PMCID: PMC4470049.
28	Patrocínio LG, Patrocínio TG, Barreto DM, Subhan YS, Patrocínio JA. Evaluation of lateral crural steal in nasal tip surgery. JAMA Facial Plast Surg. 2014 Nov-Dec;16(6):400-4. doi: 10.1001/jamafacial.2014.486. PMID: 25171047.
29	Alharethy S, Aldaghri F, Mesallam TA, Farahat M, Bukhari MA. Nasal bone length in Saudi rhinoplasty: a clinical-radiological study. Ann Saudi Med. 2014 Jan-Feb;34(1):65-7. doi: 10.5144/0256-4947.2014.65. PMID: 24658556; PMCID: PMC6074934.
30	Turkmani MG, De Boulle K. Excessive columellar show: causes, presentations and a new therapeutic approach with onabotulinum toxin A. Dermatol Surg. 2013 Mar;39(3 Pt 1):464-7. doi: 10.1111/dsu.12118. Epub 2013 Jan 17. PMID: 23331312.
31	Al-Arfaj AA, Al-Swiahb JN, Al-Harthy S, Al-Essa M. Nasal packing in cosmetic and functional nasal surgery. Saudi Med J. 2008 Jul;29(7):994-7. Erratum in: Saudi Med J. 2008 Oct;29(10):1523. PMID: 18626528.
32	Al-Qattan MM. Augmentation of the nasal dorsum with autogenous costal cartilage using the "edge-on" technique. Ann Plast Surg. 2007 Dec;59(6):642-4. doi: 10.1097/01.sap.0000258952.61173.12. PMID: 18046145.
33	Bizrah MB. Rhinoplasty for Middle Eastern patients. Facial Plast Surg Clin North Am. 2002 Nov;10(4):381-96. doi: 10.1016/s1064-7406(02)00032-9. PMID: 15062299.
34	Bizrah MB. Modified vertical dome division without delivery of the lateral crus. Arch Facial Plast Surg. 2002 Jul-Sep;4(3):198-9. doi: 10.1001/archfaci.4.3.198. PMID: 12167081.
35	Bafaqeeh SA, Al-Qattan MM. Simultaneous open rhinoplasty and alar base excision: is there a problem with the blood supply of the nasal tip and columellar skin? Plast Reconstr Surg. 2000 Jan;105(1):344-7; discussion 348-9. doi: 10.1097/00006534-200001000-00054. PMID: 10627004.
36	Bafaqeeh SA, Al-Qattan MM. Open rhinoplasty: columellar scar analysis in an Arabian population. Plast Reconstr Surg. 1998 Sep;102(4):1226-8; discussion 1229. PMID: 9734449.
37	van den Berg AA. The prophylactic antiemetic efficacy of prochlorperazine and ondansetron in nasal septal surgery: a randomized double-blind comparison. Anaesth Intensive Care. 1996 Oct;24(5):538-45. doi: 10.1177/0310057X9602400505. PMID: 8909662.
38	Van den Berg AA, Savva D, Honjol NM, Prabhu NV. Comparison of total intravenous, balanced inhalational and combined intravenous-inhalational anaesthesia for tympanoplasty, septorhinoplasty and adenotonsillectomy. Anaesth Intensive Care. 1995 Oct;23(5):574-82. doi: 10.1177/0310057X9502300508. PMID: 8787257.
39	Mathar MI, Shamsudeen SM. Maxillofacial rehabilitation of nasal defect with nasal prosthesis using donor method: A case report. Niger J Clin Pract. 2020 Jul;23(7):1022-1025. doi: 10.4103/njcp.njcp_657_19. PMID: 32620735.
40	Farook TH, Jamayet NB, Abdullah JY, Rajion ZA, Alam MK. A systematic review of the computerized tools and digital techniques applied to fabricate nasal, auricular, orbital and ocular prostheses for facial defect rehabilitation. J Stomatol Oral Maxillofac Surg. 2020 Jun;121(3):268-277. doi: 10.1016/j.jormas.2019.10.003. Epub 2019 Oct 11. PMID: 31610244.
41	Nuseir A, Hatamleh MM, Alnazzawi A, Al-Rabab'ah M, Kamel B, Jaradat E. Direct 3D Printing of Flexible Nasal Prosthesis: Optimized Digital Workflow from Scan to Fit. J Prosthodont. 2019 Jan;28(1):10-14. doi: 10.1111/jopr.13001. Epub 2018 Nov 29. PMID: 30461125.
42	Kinouchi N, Horiuchi S, Yasue A, Kuroda Y, Kawai N, Watanabe K, Izawa T, Hashimoto I, Hassan AH, Tanaka E. Effectiveness of presurgical nasoalveolar molding therapy on unilateral cleft lip nasal deformity. Saudi Med J. 2018 Feb;39(2):169-178. doi: 10.15537/smj.2018.2.21020. PMID: 29436566; PMCID: PMC5885094.
43	Elsayed M, Alosaimy RA, Ali NY, Alshareef MA, Althqafi AH, Rajab MK, Assalem AS, Khiyami AJ. Nerve Block for Septorhinoplasty: A Retrospective Observational Study of Postoperative Complications in 24 Hours. Cureus. 2020 Feb 12;12(2):e6961. doi: 10.7759/cureus.6961. PMID: 32190509; PMCID: PMC7067574.
44	Mansour HA. Repair of nasal septal perforation using inferior turbinate graft. J Laryngol Otol. 2011 May;125(5):474-8. doi: 10.1017/S0022215110002744. Epub 2011 Jan 27. PMID: 21269557.
45	Siclovan HR, Jomah JA. Injectable calcium hydroxylapatite for correction of nasal bridge deformities. Aesthetic Plast Surg. 2009 Jul;33(4):544-8. doi: 10.1007/s00266-008-9281-0. Epub 2008 Dec 9. PMID: 19067034.
46	Alhussain OH, Alhussain GO, Rajkhan AA, Suhluli AJ, Alharbi SJ, Sherwani AA, Alzamil AF, Mohammed MA. Efficacy of autologous vs. homologous costal cartilage grafts in dorsal augmentation rhinoplasty: a systematic review and meta-analysis. Annals of Medical and Health Sciences Research. 2020;10(5).
47	Alshehri AA. Perioperative Dexamethasone and Post-Rhinoplasty Edema: A Prospective Observational Study. International Journal of Otolaryngology and Head & Neck Surgery. 2021 Dec 7;11(1):1-1.
48	Abdullah AA. Quality of Work among Saudi Patients before and After Rhinoplasty. Saudi J Med.;6(11):385-90.
49	Aldhabaan SA, Hudise JY, Aldosari BF. Comparison Between Using Monocryl Suture and Polypropylene Suture in Closure of Alar Base Excision in Rhinoplasty. Plastic Surgery. 2020 Nov 23:2292550320969651.
50	Arfaj AM, Obeid AA, Subhan Y. Excessive Alar Base Resection in Rhinoplasty: How to Deal With It Once Recognized Intraoperatively?. Plastic Surgery Case Studies. 2017 Jun 30;3:2513826X17716453.
51	Lorenz KJ, Schmidt S, Abuzinadah HR, Albar NY. The Role of Fibrinogen-Thrombin Coated Collagen Sponge (TachoSil©) in Rhinoplasty: Our Experience with 78 patients. Laryngo-Rhino-Otologie. 2021 May;100(S 02).
52	Howldar S, Fida A, Allinjawi O, Zaqzoog F, Qurban G. Long-term cosmetic and functional outcomes of rhinoplasty: A cross sectional study of patients’ satisfaction. Saudi Journal of Otorhinolaryngology Head and Neck Surgery. 2018 Jan 1;20(1):1.
53	Aldosari B. Is nasal skin thickness a prognostic indicator to postoperative edema and ecchymosis?. Ear, Nose & Throat Journal. 2021 May;100(4):NP206-9.
54	: Amani A Obeid. Rhinoplasty in Thick Skinned Patients, is there a Role for the Dermatologist?. JOJ Dermatol & Cosmet. 2019; 2(1): 555579. DOI: 10.19080/JOJDC.2018.02.55557
55	Alghonaim Y, Alobaid F, Alnwaiser J. The Nasal Tip Rotation After Primary Rhinoplasty Using Columellar Strut Graft. Cureus. 2021 Mar 28;13(3).
56	Alharethy S, Al-Quniabut I, Jang YJ. Anthropometry of Arabian nose using computed tomography scanning. Annals of Saudi Medicine. 2017 Mar;37(2):144-7.
57	ALBAR N, ABUZINADAH H, LORENZ K. Fibrinogen-Thrombin Coated Collagen Sponge (TachoSil®) Usage in Rhinoplasty: Our Experience. ENT Updates. 2020 Dec 1;10(3):444-56.
58	Suwayyid W, Alshbini M, Alotaibi S, Habib L, Alotaibi S, Abuaziz R, Alkubaisy Y. The public view of media impact on seeking cosmetic surgeries in Saudi Arabia: A cross sectional study. Medical Science. 2020;24(106):4689-97.
59	Alharethy SE. The ideal aesthetic nasal dorsum in the Saudi population. Saudi Med J. 2013 Sep 1;34(9):920-2.
60	Al Harthy S, Al Arfaj A. Columellar scars in middle eastern patients are they exceptionally obvious?. Saudi J Otorhinolaryngol Head Neck Surg 2012;14:92-3
61	Aldosari BF, Alkarzae M, Almuhaya R, Aldhahri R, Alrashid H. Effect of media on facial plastic surgery in Saudi Arabia. Cureus. 2019 Nov 25;11(11).
62	Alkarzae M, Aldosari B, Alalula L, Almuhaya R, Alawadh I. The Effect of Selfies on Cosmetic Surgery. ENT Updates. 2020 Apr 1;10(1):251-60.
63	Alotaibi AD. The common complications after septoplasty and septorhinoplasty: A report in a series of 127 cases. International Journal of Otolaryngology and Head & Neck Surgery. 2017 Nov 30;6(06):71.
64	Aly MS, Raza SA, Al-Zuraiqi B, Awad BI, Alghamdi AS, Ibrahim MS, Aldhahwani BM, Shahbahi R, Alzaidi AA, Althobaiti IA, Marglani O. The outcome of temporalis fascia versus diced cartilage wrapped in temporalis fascia for dorsal augmentation in septorhinoplasty. Saudi Journal of Otorhinolaryngology Head and Neck Surgery. 2017 Jul 1;19(2):58.
65	Alarfaj A, AlJasser A, Subhan Y. Orbital emphysema as a rare complication of septorhinoplasty. J Rhinol-Otol. 2014;2:14-7.
66	Alanazy S, Aldriweesh T. Identical Twins Rhinoplasty; Twice the Challenge.
67	Aldhabaan S, Hudise JY, ALqarny M, Alarfaj A. Catgut versus polypropylene sutures for transcolumellar incision closure in open rhinoplasty: a retrospective cohort study. Cureus. 2020 Aug 15;12(8).
68	Ragab A, El Shamaa H, Ibrahim M. Dexmedetomidine, morphine, propofol vs midazolam, morphine, propofol for conscious sedation in rhinoplasty under local anesthesia. A prospective, randomized study. Egyptian Journal of Anaesthesia. 2013 Jul 1;29(3):181-7.
69	Eldin MS, Natto LY, Khiyami AJ. The surgical combination between functional endoscopic sinus surgery and septorhinoplasty as a procedure for nasal polyps treatment: a case report.
70	Bafaqeeh SA. Lateral nasal osteotomies: does the creation of a periosteal tunnel influence the degree of postoperative ecchymosis?. Canadian Journal of Plastic Surgery. 2000 Jun;8(3):101-2.
71	Elsayed M, Alghamdi AS, Khan M, Habibullah A, Alshareef MA, Senan H, Hazazi S, Alqurashi AA, Alosiami FG. Causes, Prevention, and Correction of Complications of Primary and Revision Septorhinoplasty. Cureus. 2021 Dec 21;13(12).
72	Waseem Radwan D, Almutairy GF, Aldayel NS, Althowaini NA, Alhabib TA. Female lip esthetic and its effect on facial attractiveness in Saudi Arabia. Int J Esthet Dent. 2021;16:2-9.
73	Yousef-Mian M. Repair of nasal septal perforation. American Journal of Rhinology. 1997 Jan;11(1):35-40.
74	Al-Arfaj A, Al-Qattan M, Al-Harethy S, Al-Zahrani K. Effect of periosteum elevation on periorbital ecchymosis in rhinoplasty. Journal of Plastic, Reconstructive & Aesthetic Surgery. 2009 Nov 1;62(11):e538-9.
75	FARAG MF, AL-BIZRAH MB. Modified Vertical Dome Division Versus Standard Tip Refinement Technique, a Comparative Retrospective Study.
76	Alghonaim YA, Solomon PD. Bilateral lower-lip foreign body granuloma secondary to hyaluronic acid injection. Plastic Surgery Case Studies. 2016 Jun;2(2):16-7.
77	Eldin MS, Alomari AA, Aljuaid FM, Almalki FK, Altowairqi AF, Halawani MA, Alsulami AA. Radiofrequency-assisted septorhinoplasty: a retrospective single descriptive study among Saudi patients.
78	Hudise JY, Aldhabaan SA, Aldosari BF. Complications of the nasal dorsum reconstruction using autologous or alloplastic grafts: evidence from systematic review and meta-analysis. Brazilian Journal of Otorhinolaryngology. 2020 Aug 8.
79	Abdulrauf BM. An ultimate method for cleft nasal deformity correction at primary lip surgery: Innovative concepts and review. Arch Oral Maxillofac Surg. 2020;3:36-49.
80	Kundi I. Cephalometric Soft tissue standard and gender dimorphism in nasal prominence estimated by holdaway's analysis in patients visiting college of dentistry, aljouf university. J Contemp Dent Pract. 2017 Feb 1;18(2):152-5.
